# Missed Opportunities in Tuberculosis (TB) Prevention in Pediatric Population: A Retrospective Observational Study From North India

**DOI:** 10.7759/cureus.104880

**Published:** 2026-03-09

**Authors:** Sangeeta Kumari, Aarti Agarwal, Manveer Singh, Komal Grover, Jagdish Chandra, Tribhuvan Pal Yadav

**Affiliations:** 1 Pediatrics, Employees' State Insurance Corporation (ESIC) Medical College and Hospital, Faridabad, Faridabad, IND; 2 Paediatrics, Employees' State Insurance Corporation (ESIC) Medical College and Hospital, Faridabad, Faridabad, IND; 3 Preventive Medicine, Sudha Medical College, Kota, IND; 4 Microbiology, Employees' State Insurance Corporation (ESIC) Medical College and Hospital, Faridabad, Faridabad, IND; 5 Pediatrics, National Thalassemia Welfare Society, Delhi, IND; 6 Pediatric Rheumatology, Employees' State Insurance Corporation (ESIC) Medical College and Hospital, Faridabad, Faridabad, IND

**Keywords:** and tuberculosis preventive treatment, contact tracing, family surveillance, household contacts, ntep, pediatric tuberculosis, public health care

## Abstract

Background

Contact investigation, particularly Household contact tracing (HHC), is a cornerstone of tuberculosis* *(TB) control programs globally. However, the implementation of contact tracing and TB preventive treatment (TPT) in India remains suboptimal, especially in the pediatric age group. This study aimed to determine the missed opportunities of disease identification and prevention among pediatric TB cases with a documented history of household exposure to a TB index case.

Primary objective

To determine the proportion of microbiologically confirmed pediatric TB cases with documented household exposure in whom contact tracing was not performed.

Methodology

This retrospective observational study was conducted among pediatric TB patients of age up to 12 years with microbiological evidence of the disease, enrolled from the pediatric and microbiology departments of the institute and five other satellite hospitals during the study period of two years from Jan 2023 to December 2024.

Results

Of 1,375 presumptive pediatric TB cases, 133 [9.67%; 95% confidence interval (CI): 8.1-11.2} were microbiologically confirmed, of which 132 were enrolled. Household TB exposure was documented in 71 cases (53.8%; 95% CI: 45.3-62.3). Exposure to pulmonary TB index cases occurred in 58 cases (43.9%; 95% CI: 35.5-52.4) and 139 of 169 pediatric household contacts (82.2%; 95% CI: 76.4-88.0). Contact tracing was performed for 30 of 58 exposed cases (51.7%; 95% CI: 38.9-64.5) and 48 of 139 pediatric household contacts (34.5%; 95% CI: 26.6-42.4). TPT was initiated in only 5 of 34 eligible children (14.7%; 95% CI: 2.8-26.6). Rifampicin resistance status was indeterminate in 36 of 132 cases (27.3%; 95% CI: 19.7-34.9), and resistance to it was observed in 5 out of 100 (5%) cases.

Conclusion

Significant programmatic gaps exist in pediatric TB prevention, particularly in contact tracing, TPT uptake, and drug-resistance testing. While these findings highlight missed preventive opportunities, further analytical studies are needed to identify determinants and evaluate their impact on transmission dynamics.

## Introduction

India’s tuberculosis control efforts began in the early 20th century with sanatoria and dispensaries, followed by the introduction of radiographic diagnosis and a nationwide Bacillus Calmette-Guérin (BCG) vaccination campaign in 1951 [1]. The National Tuberculosis Programme (1962) laid the foundation for organized control but achieved limited impact, leading to the Revised National Tuberculosis Control Programme (1997), which implemented the directly observed treatment short course (DOTS) strategy and expanded free tuberculosis (TB) services [2-4]. India’s current National Tuberculosis Elimination Programme, guided by the National Strategic Plan (2017-2025), aims to eliminate TB through the pillars of four components, viz.: Detect, Treat, Prevent, and Build [5].

Under the Prevent pillar, systematic and accountable contact tracing and investigation are emphasized to identify secondary TB cases, along with active screening of high-risk groups and provision of tuberculosis preventive treatment (TPT). This approach follows a cascade-of-care model in which at-risk populations are proactively identified, screened for active disease, and, after exclusion of TB, offered TPT as part of a continuum of care to improve individual outcomes and reduce transmission [6].

These preventive strategies are particularly critical for pediatric TB, which remains a major public health concern in India. In 2023, TPT was initiated in 186,997 (60% of estimated eligible) household contacts aged <5 years and 840,474 (26% of estimated eligible) contacts aged ≥5 years, along with 443,253 people living with human immunodeficiency virus (HIV), highlighting ongoing efforts to reduce disease risk in high-priority groups, but this is far off from the set targets of 90%. [6]. In 2019, only 49% of all notified TB patients underwent household contact assessments, and 78% of child contacts identified received TPT. Overall initiation and completion rates remain low [7]. Although children account for an estimated 31% of the global pediatric TB burden, they constitute only 6-7% of cases reported annually under the National Tuberculosis Elimination Programme (NTEP), reflecting a substantial detection gap. Similarly, the reported proportion of multidrug-resistant (MDR)/rifampicin-resistant (RR)-TB among children under 14 years remains around 3%, suggesting under-recognition of the true disease burden [8].

Several factors contribute to these gaps in contact tracing and TPT coverage. These include a lack of awareness among the general population, the COVID-19 pandemic, a lack of continuous motivation among healthcare workers, suboptimal resources, and the long duration of prophylaxis. Additionally, poor compliance with the TPT and the programmatic limitation of providing TPT to the contacts of pulmonary TB cases only are possibly adding to the challenges.

In light of the above, this study aimed to determine the missed opportunities of disease identification and prevention among pediatric TB cases with a documented history of household exposure to a TB index case.

Thus, the primary objective was to determine the proportion of microbiologically confirmed pediatric TB cases with documented household exposure in whom contact tracing and appropriate screening were not performed at the time of index case diagnosis. The secondary objectives were: 1) to determine the socio-demographic profile of enrolled cases, 2) to assess the status of TPT uptake among eligible pediatric contacts [cases and other pediatric household contact tracing (HHC)] at the time of index case diagnosis, and 3) to evaluate the diagnostic and drug-resistance profiling of the cases.

## Materials and methods

This retrospective cross-sectional observational study was conducted at a medical college in North India, which caters only to ESI (Employees’ State Insurance) insured persons, from Jan 2023 to Dec 2024 in the department of Paediatrics and Microbiology. The catchment areas for TB cases for the institution were six Employees’ State Insurance Corporation (ESIC) hospitals of Haryana, Delhi, and Uttar Pradesh, which covered an area of approximately around 70km. From all the centres, presumptive TB cases were referred for the microbiological diagnosis to this medical college. The number of presumptive cases and contact details of all Paediatric TB patients up to 12 years of age with microbiological evidence of the disease were extracted from the microbiology database and invited telephonically for in-person inquiry in the pediatric department. Patients were enrolled after obtaining desired consent from the parent(s)/caregivers and applying suitable inclusion/exclusion criteria, which are stated below-

Inclusion criteria

Inclusion criteria consisted of children aged ≤12 years, bacteriologically confirmed TB (microscopy, culture, or GeneXpert), diagnosed during the study period, and consent from the parent/guardian.

Exclusion criteria

Exclusion criteria included clinically/radiologically diagnosed TB without microbiological confirmation and population >12 years of age.

A standardized call protocol with up to three attempts on different days was used to minimize non-response. Data were collected using a pre-designed structured proforma developed based on NTEP indicators, namely sociodemographic details, Nikshay ID, clinical profile, household exposure history, contact-tracing practices, TPT eligibility, TPT initiation, drug-resistance reports of the cases, index case(s), and other pediatric HHC (when applicable). The index case information and details of contact investigation were inquired from parents first, verified later on from medical records, DOTS cards, and the Nikshay portal. Nikshay is the Government of India’s web-based TB surveillance system under the National Tuberculosis Elimination Programme (NTEP). Nikshay integrates data from public and private health sectors, enabling real-time notification, treatment monitoring, drug-resistance reporting, and contact tracing documentation [9].

**Figure 1:**
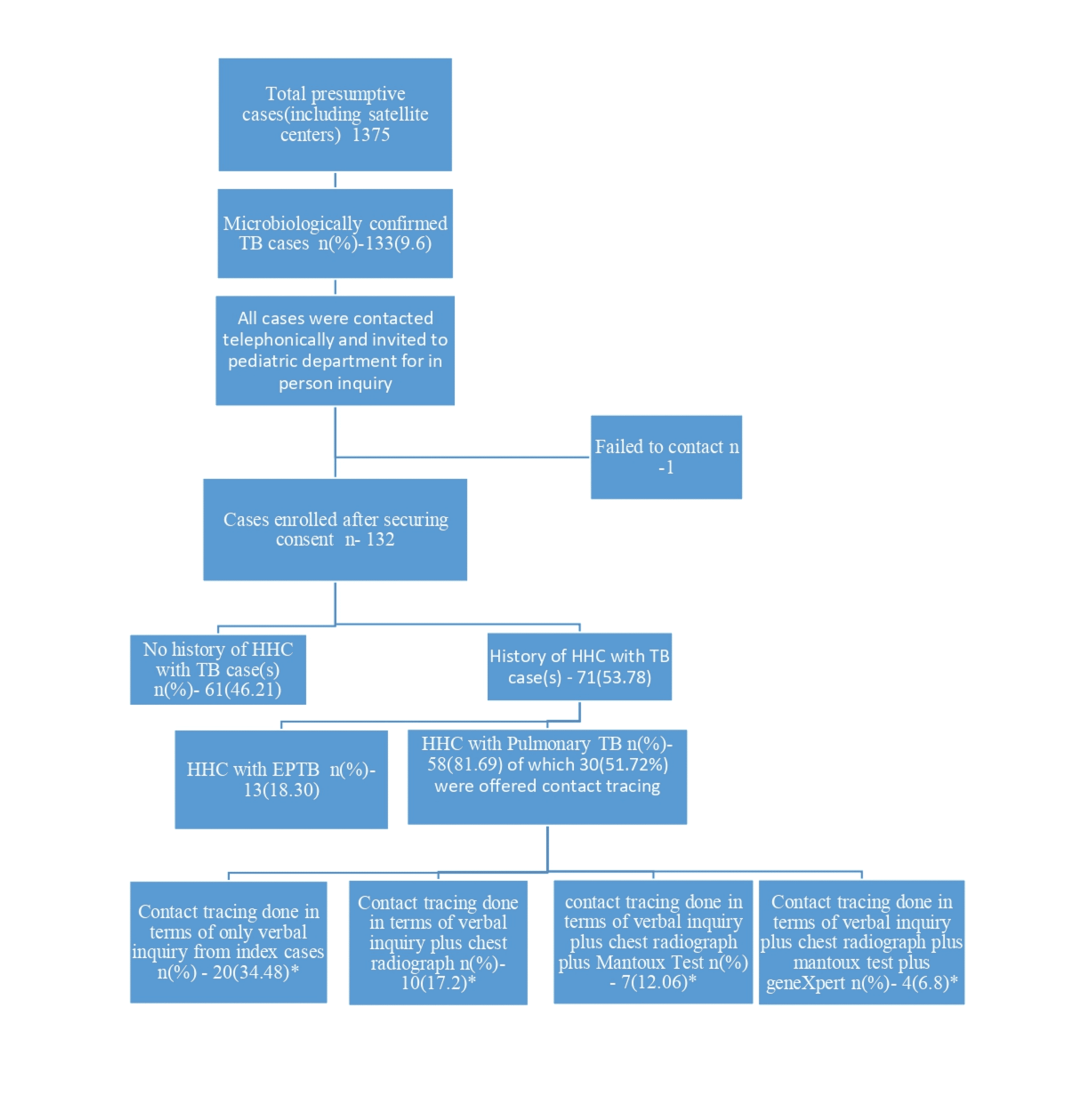
Flow chart of the case enrolment and major findings of the study. *overlapping data. Contact tracing in any form was offered to only 30 out of 58 cases with a history of household contact tracing (HHC) to a pulmonary TB patient (index case).

Socioeconomic status (SES) categorisation was done using the updated Kuppuswami scale [10]. Collected data was transferred to excel spreadsheet, hiding the patient’s identity in any form by using codes for data for record maintenance and data analysis. The flow chart of the study is drawn in Figure 1.

Institutional Ethical Committee approval was obtained with EC File number- 134X/11/13/2024-IEC/DHR/136 before the commencement of the study. Standard definitions used in NTEP were used in the study to define ‘contact tracing’, ‘household contact’, ‘close contact’ ‘TB Preventive Treatment’, ‘index case’, ‘presumptive TB’, ‘pulmonary TB’, ‘extrapulmonary TB’, etc. Other operational definitions used in the study are described in the following part of this section.

All data recorded in a pre-designed proforma (Appendix 1) was entered into a pre-coded Microsoft Excel (Microsoft Corporation, Redmond, USA) database. Data entry accuracy was verified by cross-checking 10% of records randomly by an independent investigator before importing into SPSS version 26.0 (IBM Corp., Armonk, NY, USA) for analysis. Continuous variables were expressed as mean ± SD. Categorical variables were expressed in frequencies & percentages. Proportions related to household contact history, contact tracing, radiographic screening, TPT initiation, and rifampicin resistance were calculated using variable-specific denominators.

Proportions were reported with 95% confidence intervals. Chi-square or Fisher’s exact test was applied for comparative analysis. A p-value of <0.05 was considered significant. Given the descriptive design, no causal inference was attempted. There were no missing data for the variables included in the final analysis, as data completeness was ensured at the time of collection.

Data handling and variable definitions

Each participant was assigned a unique coded identifier to maintain confidentiality and to prevent duplication during analysis of overlapping datasets (i.e., pediatric TB cases who were also documented household contacts. Variables were operationalized as follows: Age was analysed as a continuous variable (mean ± standard deviation). The type of tuberculosis was classified as pulmonary, extrapulmonary, or disseminated TB according to standard NTEP definitions.

Contact tracing practices were categorized hierarchically (used for study purposes) based on modality and included verbal enquiry alone, verbal enquiry + chest radiograph, verbal enquiry + radiograph + Mantoux test, verbal enquiry + radiograph + Mantoux test + GeneXpert, drug sensitivity testing - rifampicin sensitive, resistant, or indeterminate.

Handling of overlapping data

Pediatric TB cases who also had documented household exposure were included in both disease-characteristic analysis and contact tracing cascade evaluation; however, separate denominators were maintained for each analytical component to avoid inflation of proportions.

Recall bias was minimized through cross-verification of exposure history using programmatic records. Standard NTEP operational definitions were applied uniformly to reduce misclassification bias. Potential selection bias due to the inclusion of ESIC beneficiaries and microbiologically confirmed cases only is acknowledged in the limitations.

The analytic framework followed sequential stages: identification of microbiologically confirmed paediatric TB cases, documentation of household exposure, assessment of contact tracing practices, evaluation of screening modalities used, determination of TPT eligibility, TPT initiation, and assessment of drug resistance profiling. This cascade model allowed identification of attrition points representing missed preventive opportunities.

Operational definitions

Family Surveillance or Contact Investigation

A systematic process of identifying, screening, evaluating, and managing contacts of TB patients to detect active TB and provide preventive treatment.

Household Contact

A household contact was defined as any child residing in the same household and sharing living space with an index TB patient during the infectious period, in accordance with NTEP guidelines.

Contact Tracing

Any systematic evaluation of contacts at the index case diagnosis.

Adequate Screening

use of ≥1 diagnostic modality beyond verbal inquiry. 

Missed Opportunity

Eligible pediatric contact not screened and/or not initiated on TPT.

TPT Eligibility

Contacts <5 years exposed to pulmonary TB.

## Results

One thousand three hundred and seventy five presumptive pediatric TB patients were subjected to microbiological evaluation, of whom 133 [9.67%; 95% confidence interval (CI): 8.1-11.2] were positive for Mycobacterium tuberculosis; of these, 132 cases who gave consent were enrolled in the study (Figure 1).

The mean age of the participants was 8.45 ± 3.53 years. Cases and pediatric household contacts (HHC) under five years were 22 (16.7%; 95% CI: 10.3-23.1) and 34 (20.1%; 95% CI: 14.1-26.1), respectively. Other sociodemographic data are depicted in Table 1.

**Table 1:** Sociodemographic profile and type of TB among the cases. *Inclusive of four cases of congenital TB. SD: standard deviation, SES: socioeconomic status, TB: tuberculosis, CNS: central nervous system.

Variable	Number	Categories	Frequency n (%)	95% CI
Age in years (Mean ± SD)	132	Mean±SD	8.45 ± 3.53	-
Sex	132	Male	69(52.3)	-
		Female	63(47.7)	-
Father's Education	132	Below 10th Standard	69(52.3)	43.8–60.8
		Above 10th Standard	63(47.7)	39.2–56.2
Father's Occupation	132	Unskilled Worker	128(97.0)	94.1–99.9
		Skilled Worker	4(3.0)	0.1–5.9
Mother's Education	132	Below 10th Standard	92(69.0)	61.9–77.5
		Above 10th Standard	40(30.3)	22.5–38.1
Mother's Occupation	132	Housemaker	130(98.5)	96.4–100
		Working	2(1.5)	0–3.6
Overcrowding	132	Absent	25(18.9)	12.2–25.6
		Present	107(81.1)	74.4–87.8
SES (Modified Kuppuswamy)	132	Upper Lower and Lower	127(96.2)	92.9–99.5
		Lower Middle	5(3.8)	0.5–7.1
Clinical presentation				
Type of Tuberculosis	Total	Frequency (n)	Percentage (%)	
Overall TB	132	132	100	-
- Extra pulmonary TB		52	39.4	31.0–47.8
- Pulmonary TB		53	40.2	31.8–48.6
- Disseminated TB*		27	20.5	13.6–27.4
Affected organ/sites of TB				
- CNS TB		19	14.3	8.4–20.4
- Lymph Node TB		38	28.8	21.1–36.5
- Pleural TB		25	18.9	12.2–25.6
- Abdominal TB		31	23.5	16.3–30.7
- Genital TB		1	0.8	0–2.2
- Skeletal Muscular TB		6	4.5	1.0–8.0

All cases were subjected to GeneXpert test in either gastric lavage, sputum, pleural fluid, cerebrospinal fluid (CSF), or ascetic fluid, wherever applicable. Sputum microscopy, other bodily fluid microscopy, chest x ray and Mantoux test was done in 129(97.7%), 119(90.2%), 130(98.5%) and 130(98.5%) cases. Among the cases, 100 of 132 (75.8%; 95% CI: 68.5-83.1) were GeneXpert positive. Sputum microscopy was positive in 19 of 129 cases (14.7%; 95% CI: 8.6-20.8), and microscopy of fluids other than sputum was positive in 55 of 119 cases (46.2%; 95% CI: 37.2-55.2). On review of chest x-ray from the medical records of cases, chest -ray findings were indicative of TB infection in 105 of 130 cases (80.7%; 95% CI: 73.9-87.5), of which 70 (53.8%; 95% CI: 45.2-62.4) exhibited changes strongly suggestive of TB infection as per NTEP. The Mantoux test was positive in 84 of 130 cases (64.6%; 95% CI: 56.4-72.8).

In drug susceptibility testing, rifampicin resistance was the only first-line resistance evaluated. Of 100 GeneXpert-positive cases, 91 (91.0%; 95% CI: 85.4-96.6) were sensitive to rifampicin, and five (5.0%; 95% CI: 0.7-9.3) exhibited resistance. The status of rifampicin resistance was indeterminate in 36 of 132 cases (27.3%; 95% CI: 19.7-34.9), including 32 cases where GeneXpert was negative.

Table 1 shows the distribution of cases by type of TB. In extra-pulmonary (EP) TB, lymph node TB accounted for the majority of cases. The table shows the frequency of affected organ/site, which is not mutually exclusive. Overlapping categories were observed, as multiple anatomical sites or organ involvement occurred as part of disseminated TB. Four cases (3.0%) of congenital TB were found among these 132 cases.

Household TB exposure was documented in 71 of 132 cases (53.8%; 95% CI: 45.3-62.3). One hundred and sixty-nine children (including 132 cases) were exposed to these 71 index cases. Among index cases, 58 of 71 (81.7%; 95% CI: 72.7-90.7) had pulmonary involvement (pulmonary or disseminated TB), and 139 of 169 children (82.2%; 95% CI: 76.4-88.0) were exposed to those 58 pulmonary household index cases.

Contact tracing among identified pediatric household contacts was similar between public (52%) and private (48%) sectors. Overall, only five of 48 children (10.4%, 95% CI: 3.4-22.7) were started on TPT. Sector-wise, three children (6.3%, 95% CI: 1.3-17.2) linked to public sector index cases and two children (4.2%, 95% CI: 0.5-14.3) linked to private sector index cases received preventive therapy as depicted in Table 2. The place of TB diagnosis in index cases, status of family surveillance, and TPT uptake are shown in Table 2. Table 3 shows the proportion and nature of contact tracing offered at the time of index case diagnosis.

**Table 2:** Tuberculosis diagnosis, contact tracing and TPT initiation at public and private sectors. The Chi-square test for index case distribution among the public and private sectors revealed a p-value of < 0.001, which is very significant statistically and suggestive of majority of index cases were diagnosed in the public sector. The TPT initiation among the public and private sectors has shown no significance (p-value=1). CI: confidence interval, TPT: tuberculosis preventive treatment, HHC: household contact tracing.

Centre Type	Index cases diagnosed n (% / 95% CI)	Contact tracing among paediatric 48 of 139 HHC n (% / 95% CI)	TPT initiation among 48 screened paediatric HHC n (% / 95% CI)
Public	57 (80.3% / 69.6–88.1)	25 (52.1% / 37.4–66.5)	3 (6.3% / 1.3–17.2)
Private	14 (19.7% / 11.9–30.4)	23 (47.9% / 33.5–62.6)	2 (4.2% / 0.5–14.3)
Total	71 (100%)	48 (34.5 %)	5 (10.4% / 3.4–22.7)

**Table 3:** Household contact tracing & screening modality. The above table showing the inadequate active case finding interventions were offered at the time of index case diagnosis.

Category	Frequency n (%) among 169 HHC	95% CI
Family Surveillance	48(28.4)	21.6–35.2
Verbal enquiry	48(28.4)	21.6–35.2
Chest X-ray	21(12.4)	7.4–17.4
Mantoux Test	12(7.1)	3.2–11.0
GENEXPRT	8(4.7)	1.5–7.9
Sputum microscopy	8(4.7)	1.5–7.9

The most common modality used for contact tracing was verbal inquiry regarding symptoms among household members, including children. Ninety-one of 139 eligible pediatric household contacts (65.5%; 95% CI: 57.6-73.4) did not undergo any form of contact tracing.

## Discussion

India’s National Strategic Plan (NSP) 2017-2025 under NTEP was structured around the pillars of Detect, Treat, Prevent, and Build to end TB by 2025, emphasizing early diagnosis, systematic contact investigation, and provision of TPT for high-risk group including paediatric contacts [6]. This study provides descriptive evidence of the underachievement of the target set for prevention strategies under NTEP.

Bacteriological confirmation in only 9.67% of the 1375 presumptive TB cases of the pediatric cohort reflects the diagnostic constraints, such as availability of sputum, difficulty in extracting gastric lavage, amount and type of sample availability in addition to the paucibacillary nature of the pediatric disease [11]. It might be a strong reason to expand the plethora of diagnostic tools at the national level, e.g., inclusion of computed tomography (CT) chest and bronchio-alveolar lavage (BAL).

**Figure 2:**
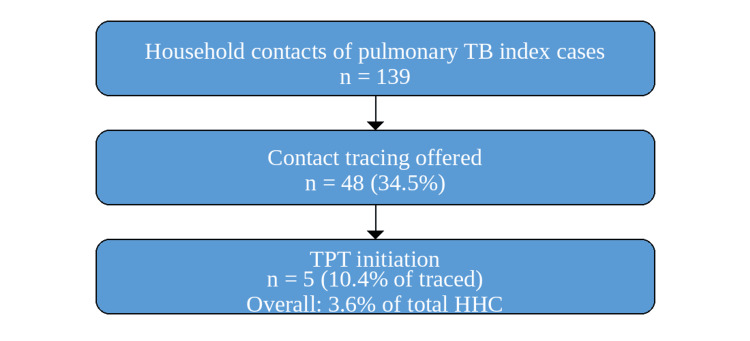
Flow diagram showing household contact screening and TPT initiation. TB prophylaxis treatment initiation was strikingly low in the studied population. TPT: tubercolosis preventive treatment.

Household contact history with any form of TB was identified in 169 children, of which 139 were exposed to pulmonary TB cases, including 71 enrolled cases; however, contact tracing was undertaken for only 48 (34.5%) eligible paediatric household contacts (Figure 2), including 30 (42.2%) exposed cases, which is substantially below national expectations [6]. Contact investigation relied largely on verbal enquiry, with chest radiography performed in only 10 of 58 (17.2%) children exposed to pulmonary TB cases (Table 3).

A retrospective study from Turkey has given a very important observation that pulmonary TB in the pediatric population remains asymptomatic and requires extensive investigations, including computed tomography [12]. In this study, it has been documented that an additional 34 (40%) out of 84 patients were diagnosed as pulmonary TB with the help of CT chest, highlighting the need for testing beyond routine investigations in this vulnerable category of patients. In addition, the latency period of the disease is quite variable [13]; therefore, the contact person might manifest the disease after the contact investigation. Hence, multiple time contact tracing for at risk child should be conducted. The current observations are suggestive of inadequate use of standard guidelines provided by the program. These findings highlight substantial missed opportunities for active case finding among high-risk paediatric contacts

In the present study, TPT initiation was documented in only five (14.7%) of 34 eligible children, way below the national target of 90% TPT coverage by year 2025 [6]. The national progress in view of TPT coverage remains uneven. The India TB Report 2024 documents TPT coverage of 80%, which is markedly below the target and with wide regional variation, as added by the current study. A community-based study from Faridabad reported higher adult TB prevalence than national estimates, suggesting persistent local transmission [14]. The low contact tracing and TPT uptake observed here might be due to 1) a highly mobile labour class population, 2) poor educational status of the parents leads to gaps in awareness, 3) reliance on verbal inquiry for contact tracing, and 4) potentially exacerbated during the COVID-19 pandemic, which has also been observed by Dey et al. as they reported reductions in TB service delivery at the time of the pandemic [15]. The disruption over the three years of the COVID-19 pandemic has likely contributed to an increase in pediatric TB cases that were probably missed during contact investigation in that period. Previous studies have highlighted the value of systematic contact investigation. Nair et al. reported a 5.3% TB yield among household contacts [16], and Jamil et al. demonstrated improved detection with structured contact listing [17]. Shah et al. and Nagarajan et al. showed that decentralized, protocol-driven approaches might strengthen preventive care [18, 19]. The extremely low uptake of tuberculosis preventive treatment (TPT) in eligible children represents another major missed opportunity in the cascade of paediatric TB prevention. These findings collectively support reinforcing paediatric contact tracing strategies.

There were 22 (16.7%) under five years and four (3%) congenital TB cases, suggesting ongoing community transmission. As paediatric TB generally reflects recent infection, a higher paediatric burden may indicate active transmission dynamics. Because only microbiologically confirmed cases were included, the proportion of pediatric cases that were diagnosed clinically or radiologically with missed contact tracing opportunities remained unknown. The restriction to microbiologically confirmed cases improves diagnostic specificity but might underestimate disease burden and missed opportunities. Although national targets were aimed at reducing TB incidence from 217 to 77 per lakh by 2023, incidence remained 199 per lakh, and inadequate interruption of paediatric contact transmission, as suggested by this study, might be a significant contributing factor.

Although EPTB is primarily excluded from routine contact tracing in the national program, in our study, 13 (18.3%) cases were exposed to EPTB index cases, suggesting possible undiagnosed pulmonary involvement or exposure outside the household. Among enrolled cases, 52 (39.4%) presented with EPTB (Table 1), with almost half of these cases having chest radiographic findings suggestive of TB infection with varying degrees, indicating a potential focus of the unchecked spread. However, these were all subjective observations and need more specific, objective, and probably AI (artificial Intelligence) driven tools for it. Literature from low TB burden areas has noted extensive investigations, including advanced radiological testing (CT chest), resulting in a significant number of diagnoses of the disease in the high-risk asymptomatic pediatric patient [12]. Studies from Northeast India and Chhattisgarh report similar EPTB burden, indicating potential of communicability and hence epidemiological relevance [20, 21]. Failure to recognize possible pulmonary foci in EPTB and to extend contact evaluation and tuberculosis preventive treatment (TPT) to exposed children could perpetuate silent transmission chains. Strengthening diagnostic evaluation of EPTB cases and reconsidering their role in contact tracing policies may therefore be essential steps toward improving case detection and interrupting transmission under the national TB elimination strategy. To rule out pulmonary focus in EPTB cases, more extensive, larger, and diagnostic interventional studies are needed to generate enough evidence to recommend contact tracing and TPT under the national program.

This study has reported Rifampicin resistance in 5% of paediatric cases with the help of GeneXpert test, while resistance against other first-line anti-tubercular drugs remained unknown due to the limited availability of drug-susceptibility testing. Globally, Dodd et al. have estimated that around 25,000 children develop multidrug-resistant (MDR) TB annually, often linked to transmission from MDR cases, inadequately treated adult cases, or due to inadequate treatment of themselves, underscoring the public health risk of incomplete resistance testing [22]. Although the programme recommends liquid drug sensitivity testing (DST) for all first-line anti-tubercular (ATT) drugs, the facilities for the same are quite sparse, which is reflected in the cohort very obviously. These findings highlight a major missed opportunity in paediatric TB care, which is that failure to perform comprehensive DST limits the ability to detect the full burden of drug resistance, leading to potential under-treatment, ongoing transmission of resistant strains, and poorer outcomes in children. Inadequate resistance testing also hampers effective source case identification during contact investigations, particularly when resistant TB may be circulating within households or communities. Strengthening universal and rapid DST access for paediatric TB patients across both public and private sectors is therefore critical. In this cohort, the majority of the index cases were diagnosed in the public sector settings, consistent with national trends where private sector notifications remain below 2023 targets [6]. Contact tracing, as well as TPT uptake, remained low among public and private sectors (Table 2). The observed associations should be interpreted as programmatic indicators rather than determinants of transmission.

Limitations of the study

The retrospective design of the study limits causal inference of determinants of poor preventive strategies due to a lack of analytical modelling. Inclusion of only microbiologically confirmed cases might have limited the exact representation of the disease burden and the missed opportunities. The retrospective study design is not considered the best to synthesize the strong conclusions. Inclusion of primarily ESIC beneficiaries may have introduced selection bias due to major socioeconomic skew, thereby limiting generalizability to the wider pediatric population.

## Conclusions

This study identifies important programmatic underachievement in pediatric TB prevention goals, particularly in contact tracing, TPT initiation, and drug-resistance testing. These findings highlight the need to augment the preventive strategy pillar of the programme implementation. Further prospective and analytical studies are required to identify determinants and evaluate interventions to strengthen pediatric TB control.

## Figures and Tables

**Figure 3:**
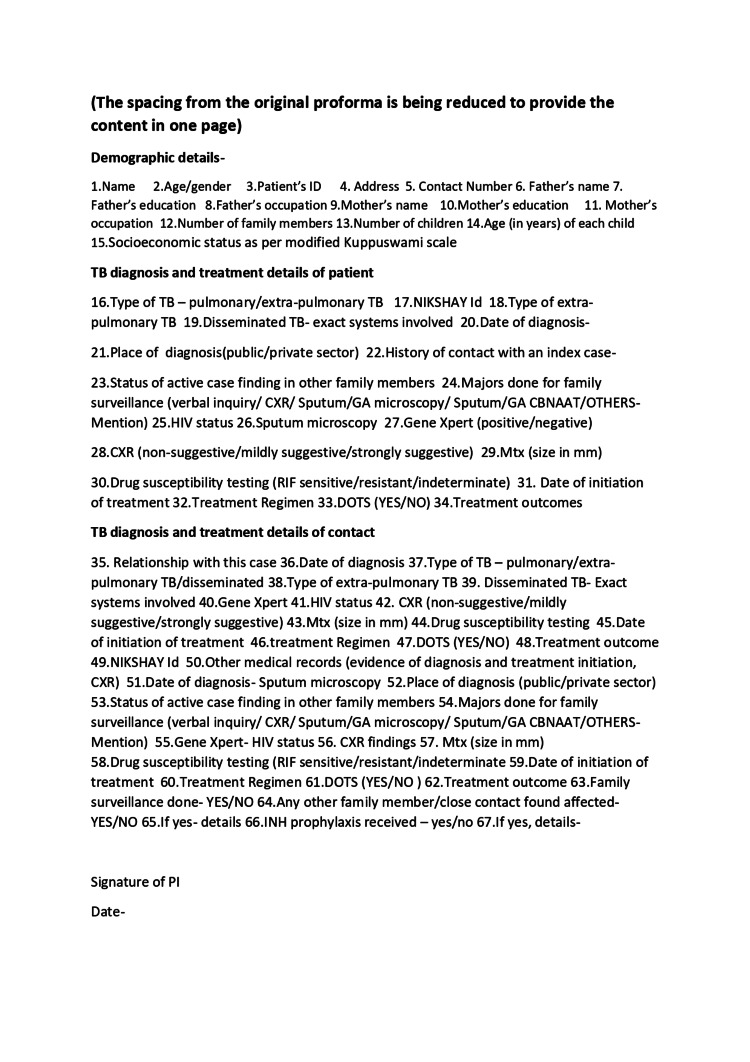
Proforma for missed opportunities in TB elimination in pediatric population in a retrospective observation from North India. TB: tuberculosis.

## Appendices

Appendix 1
